# A decade of improvements in equity of access to reproductive and maternal health services in Cambodia, 2000–2010

**DOI:** 10.1186/1475-9276-12-51

**Published:** 2013-07-09

**Authors:** Antonia Dingle, Timothy Powell-Jackson, Catherine Goodman

**Affiliations:** 1 Department of Global Health and Development, Faculty of Public Health and Policy, London School of Hygiene and Tropical Medicine, London, UK

**Keywords:** Equity, Access, Utilisation, Reproductive and maternal health services, Cambodia

## Abstract

**Introduction:**

Despite encouraging reductions in global maternal mortality rates, Millennium Development Goal (MDG) 5 on reducing maternal mortality and achieving universal access to reproductive health remains the most off-track of all MDGs. Furthermore a preoccupation with aggregate coverage statistics masks extensive disparities in health improvements between societal groups. Recent national health indicators for Cambodia highlight impressive improvements, for example, in maternal, infant and child mortality, whilst substantial government commitments have been made since 2000 to address health inequities. It is therefore timely to explore the extent of equity in access to key reproductive and maternal health services in Cambodia and how this has changed over time.

**Methods:**

Analysis was conducted on three rounds of Demographic and Health Survey data from 2000, 2005 and 2010. Outcome variables comprised utilisation of six reproductive and maternal health services – antenatal care, skilled birth attendance, facility-based delivery, postnatal care, met need for family planning and abortion by skilled provider. Four equity measures were calculated – equity gaps, equity ratios, concentration curves and concentration indices. Household assets were used to create the social-stratification variable, using principal components analysis.

**Results:**

Coverage levels of all six services improved over the decade. Coverage improvements were greatest amongst wealthier quintiles of the population, although poorer quintiles also increased use of services. Critically, inequity in service use of all services dramatically reduced over time, except for postnatal care where inequity increased slightly. However, in 2010 inequity in service use remained favouring wealthier quintiles, greatest in use of skilled birth attendance and facility-based delivery, though the magnitude of inequity was substantially reduced compared to 2000. Met need for family planning was almost perfectly equitable in 2010.

**Conclusions:**

Cambodia has made impressive improvements in overall coverage of reproductive and maternal health services over the last decade, and also in the distribution of their use across wealth quintiles. A range of pro-poor health financing and supply-side policies as well as non-health factors may have contributed to these achievements. Further research will explore specific schemes qualitatively and quantitatively to assess their impact on equity and service use.

## Background

Recent estimates suggest global maternal deaths now total approximately 287,000 per year, of which 99% occur in developing countries [1]. These figures represent a promising decline over the last three decades [2]. However, Millennium Development Goal (MDG) 5 to reduce maternal mortality by 75% by 2015 and achieve universal access to reproductive health remains amongst the most off-track of all MDGs [3]. Furthermore, consideration of only national-level statistics masks trends in the distribution of health outcomes and access to services across income groups. As the international health community considers a post-MDG agenda, with increasing focus on universal health coverage, it is essential to not only consider overall health improvements, but also how *equitable* those improvements are.

There is now a burgeoning focus on equity within the public health arena, particularly in the maternal health field [4], perhaps reflecting the priority of policymakers. The growing literature on equity in reproductive and maternal health in developing countries suggests the stark disparities in maternal mortality, morbidity and in the use of reproductive and maternal health services are the “*largest discrepancy in public health statistics*” [4]. High levels of inequity have also been documented within countries, in skilled birth attendance (SBA), facility-based deliveries (FBD) and contraceptive use by wealth quintile [5-15], education status [6,7,11,16], and urban versus rural location [10,11,14-20], with better outcomes favouring more advantaged groups. The evidence is less consistent regarding use of antenatal care (ANC) [5,6,8-11,13-15,21], and data are very limited on postnatal care (PNC) [6,11]. Globally rates of unsafe abortion have been found to be higher amongst young women, whilst there is evidence of higher complication rates and mortality from unsafe abortion amongst women of lower socio-economic status [22].

Most studies to date focus on one or two specific reproductive and maternal health services [5,7,12,14,15,18], however few assess equity in use of the whole spectrum of services, from contraception to postnatal care [6,8,16]. Furthermore the majority of the literature focuses on a single year of data, inhibiting consideration of trends in equity over time [5,6,8,11,12,14,15,17],[23]. Finally, state-of-the-art methods for measuring equity, such as concentration curves and indices [24], are applied infrequently in studies of reproductive and maternal health equity [8,9,11,23]. Rather, studies tend to report general coverage levels or equity gaps, equity ratios and odds ratios of use in richest compared to poorest wealth quintile [5,6,12,14-16,25,26]. This study attempts to address these critical gaps in the context of Cambodia.

In recent years Cambodia has made a concerted effort to address health inequities and the issue recurs as a prominent theme within many of the country’s health policies, as does a specific focus on equity in maternal and newborn health [27-30]. On the demand-side, there has been a focus on access to health services for the poor through the expansion of user fee exemptions, health equity funds (HEF), vouchers and community-based health insurance (CBHI). The most prominent of these are HEFs, which provide health insurance for the poor at tertiary and increasingly at primary levels, through a third party purchaser of health services that reimburses providers for free services provided to those identified as poor. Despite these efforts, out-of-pocket payments on healthcare in Cambodia remain high with the cost of healthcare posing a poverty trap for the country’s poor [31]. On the supply-side, policies to improve maternal and newborn health include the training of midwives, a midwifery financial incentive scheme, and a ban on home deliveries by traditional midwives.

In this context, Cambodia makes an interesting case study within which to explore health equity and how this has changed since these policies were first introduced. As such, this paper aims to assess equity in access to reproductive and maternal health services – ANC, SBA, FBD, PNC, safe abortion and met need for family planning – over a decade in Cambodia.

## Methods

### Study setting

Cambodia is one of the poorest countries in South East Asia [32]. Of the 14.8 million population, 80% is rural [33]. Between 2000–2008, an estimated 25.8% of the population were living on less than US$1 per day [34]. Poverty has decreased somewhat over the last decade due to Cambodia’s rapid economic development [35]. However Cambodia’s transitioning economy has also ushered in increasing socio-economic inequalities. Cambodia’s Gini index grew from 38 in 1994 to 44 in 2007, though the most recent estimate indicates Cambodia’s Gini index dropped again to 38 in 2008. In 2008, income inequality in Cambodia was the second highest in the Mekong region, behind Thailand [36].

### Outcomes

Six reproductive and maternal health utilisation variables were analysed: 1) use of at least four ANC visits, the World Health Organisation (WHO) recommended minimum number of visits during pregnancy [37]; 2) delivery with a skilled birth attendant, defined as a trained doctor, nurse or midwife [38]; 3) delivery in a health facility; 4) a PNC visit with a skilled birth attendant after delivery [38,39]; 5) total met need for contraception, defined as the proportion of married women or those in a union who are sexually active and fecund, who are using contraception to stop or delay childbearing [40]; and 6) provision of abortion by a trained provider, defined as a doctor, nurse, midwife or other trained mid-level (non-physician clinician), in a health facility [41].

### Data and social stratification variables

Demographic and Health Surveys (DHS) conducted in Cambodia in 2000, 2005 and 2010 were used to estimate equity in access to the six reproductive and maternal health services over the last decade. The DHS is a nationally representative household survey of men and women aged 15 to 49 years. The Cambodia DHS 2010 sampled approximately 18,700 women and 8,200 men in 15,600 households [42]. For all three surveys households were sampled from 14 individual provinces and five groups of provinces to produce 19 sampling domains, stratified into urban and rural areas [42]. Data on use of abortions, FBD and SBA was collected from survey participants using a five year recall period; specifically for FBD and SBA this related to all live births in the five years prior to the survey. Data on ANC and PNC was collected only regarding the most recent live birth in the five years prior to the survey. Data on contraception use relates to current use at the time of data collection [42-44].

Household wealth was used in the primary analysis as the social-stratifying variable across which equity in service use was estimated. For comparison, we also conducted an analysis with education as the social-stratifying variable, the results of which are reported in Additional file 1. Household wealth constructed through an asset index is argued to be a valid and reliable proxy measure for more robust indicators of wealth such as household income or consumption not contained within the DHS [45-47]. The asset index was constructed for each survey round using principal component analysis, incorporating three categories of assets – durable consumer goods (e.g. ownership of a refrigerator, television, motorbike, etc.); quality of the dwelling (e.g. roof material, floor material etc.); and access to utilities and infrastructure (e.g. main source of drinking water, type of toilet facility). All available variables in these categories in each year of the survey were included in the asset index. As DHS questionnaires varied slightly across the years there are some differences in the asset variables included in each year. Therefore the analysis was conducted using both a common set of assets from 2000 in each year, and the maximum set of assets available for each year, to explore the sensitivity of the results to changes in the set of asset variables used to construct the wealth score. The results presented here are those estimated using all available assets per year; estimates based on a common set of assets are reported in Additional file 2.

### Statistical analysis

Descriptive statistics were calculated for women in each year by health service utilisation outcome variables. Individual service coverage levels were calculated in each year by tabulating health service variables by asset wealth quintiles. A composite coverage index was also calculated, taking an equally weighted average of service coverage per wealth category for each year [23,48]. Subsequently four standard equity measures were estimated for each health service in each year – equity gaps, equity ratios, concentration curves and concentration indices [49].

The equity gap is the absolute percentage point difference in service use between the highest and lowest quintiles. The equity ratio is estimated by dividing service coverage in the highest quintile by that in the lowest. The concentration curve plots the cumulative sample population, ranked by wealth, against the cumulative proportion of health service utilisation. The diagonal line from the origin reflects perfect equality. Concentration curves lying everywhere below the line of equality reflect disproportionate service utilisation benefiting richer individuals within the population; curves lying everywhere above the line of equality reflect disproportionate service utilisation amongst poorer individuals [24,50,51].

The concentration index is calculated as twice the area between the concentration curve and the line of equality and measures the extent of inequality systematically associated with wealth. The index takes a value between −1 and 1; 0 indicates perfect equity [24,50]. The concentration index incorporates data from the whole population, is sensitive to the population distribution across socio-economic groups and takes into account the socio-economic dimension of health, ranking populations by wealth rather than health status [50]. As such it provides the richest description of inequity. Our primary equity measure, indirectly standardised concentration indices, standardising for age within the sample population [24,52], are presented for the entire country and for urban and rural subgroups. Following O’Donnell et al. (2008) we calculated the standard error and confidence intervals for each concentration index [24].

## Results

Descriptive statistics for DHS samples of women of reproductive age from 2000, 2005 and 2010 are presented in Table 1. Descriptive statistics for women by health service, by year, are presented in Additional file 3.

**Table 1:** **Summary descriptive statistics of women aged 15**-**49**, **Cambodia**, **DHS 2000**-**2010**

**Year**	**2000**		**2005**		**2010**	
**Variable**	**Mean**	**Standard deviation**	**Mean**	**Standard deviation**	**Mean**	**Standard deviation**
Age (years)	29.67	10.14	29.80	10.30	29.87	10.19
Household (people)	6.08	2.30	5.77	2.29	5.52	2.16
Urban residence	0.18	0.38	0.17	0.38	0.21	0.41
**Highest level of education**						
No education	0.28	0.45	0.20	0.40	0.16	0.37
Primary	0.55	0.50	0.56	0.50	0.49	0.50
Secondary	0.17	0.37	0.24	0.42	0.32	0.47
Higher	0.004	0.06	0.01	0.10	0.03	0.17
**Religion**						
Buddhist	0.96	0.19	0.97	0.17	0.97	0.16
Muslim	0.03	0.16	0.02	0.13	0.01	0.12
Christian	0.003	0.05	0.01	0.08	0.01	0.07
Other	0.01	0.10	0.01	0.09	0.01	0.09
**Marital status**						
Never married	0.32	0.47	0.32	0.47	0.31	0.46
Married	0.59	0.49	0.60	0.49	0.62	0.49
Widowed	0.06	0.24	0.05	0.23	0.04	0.23
Divorced	0.03	0.16	0.03	0.20	0.05	0.24
Not living together	0.01	0.07	0.02	0.17	0.01	0.16
**Husband occupation**						
Did not work	0.03	0.18	-	-	0.03	0.18
Professional/technician/manager	0.11	0.31	0.06	0.24	0.11	0.31
Clerical	0.01	0.11	0.02	0.15	0.01	0.11
Sales	0.04	0.20	0.07	0.25	0.04	0.20
Agricultural self-employed	-	-	0.48	0.50	-	-
Agricultural employee	0.66	0.47	0.11	0.31	0.66	0.47
Services	0.01	0.11	0.07	0.25	0.01	0.11
Skilled manual	0.09	0.29	0.11	0.31	0.09	0.29
Unskilled manual	0.03	0.18	0.08	0.27	0.03	0.18

N (2000) = 15,305; N (2005) = 16,617; N (2010) = 18,504.

### Trends in service use

Table 2 illustrates that coverage of reproductive and maternal health services in Cambodia has improved over the last decade, for some services substantially. The greatest increase in use was for at least four ANC visits during pregnancy, followed closely by FBD. The smallest change in coverage was in abortion with a skilled provider, though reported use of this service was already high in 2000 at 82% of all reported abortions.

**Table 2:** Summary of magnitudes of inequities by health service, Cambodia, 2000-2010

	**Year**	**N**	**Overall coverage (%)**	**Q1 coverage (%)**	**Q5 coverage (%)**	**Equity gap (Q5-Q1, % points)**	**Equity ratio (Q5/Q1)**	**Indirectly standardised concentration index (95% confidence interval)**	**Indirectly standardised concentration index rural women (95% confidence interval)**	**Indirectly standardised concentration index urban women (95% confidence interval)**
4 + antenatal care	2000	6049	9.00%	2.20%	21.50%	19.30%	9.7	0.43	0.27	0.077
								(0.16,0.70)	(0.20,0.34)	(0.07,0.84)
	2005	6075	27.00%	13.80%	51.40%	37.60%	3.7	0.28	0.12	0.69
								(0.25,0.31)	(0.08,0.16)	(0.65,0.73)
	2010	6371	57.30%	37.40%	79.47%	42.07%	2.1	0.15	-0.08	0.58
								(0.13,0.17)	(-0.12,-0.04)	(0.55,0.61)
Skilled birth attendance	2000	8729	32.20%	14.30%	66.20%	51.90%	4.6	0.33	0.23	0.64
								(0.29,0.37)	(0.19,0.27)	(0.58,0.70)
	2005	8201	43.80%	14.40%	86.80%	72.40%	6	0.35	0.22	0.66
								(0.30,0.40)	(0.18,0.26)	(0.59,0.73)
	2010	8115	68.80%	42.20%	96.80%	54.60%	2.3	0.17	-0.05	0.58
								(0.15,0.19)	(-0.08,-0.02)	(0.51,0.65)
Facility based delivery	2000	8746	10.00%	1.80%	29.20%	27.40%	16.1	0.58	0.47	0.76
								(0.52,0.64)	(0.39,0.55)	(0.71,0.81)
	2005	8201	21.20%	5.20%	56.80%	51.60%	10.8	0.50	0.34	0.78
								(0.43,57)	(0.28,0.40)	(0.68,0.88)
	2010	8138	53.10%	29.20%	82.90%	53.70%	2.8	0.22	-0.03	0.60
								(0.20,0.24)	(-0.07,0.01)	(0.53,0.67)
Postnatal care	2000	8737	54.60%	40.90%	70.50%	29.60%	1.7	0.10	0.02	0.48
								(0.08,0.12)	(-0.01,0.05)	(0.41,0.55)
	2005	60.76	67.60%	51.60%	84.60%	33.00%	1.6	0.09	-0.02	0.45
								(0.07,0.11)	(-0.05,0.01)	(0.37,0.53)
	2010	6374	73.80%	51.80%	90.20%	38.40%	1.7	0.12	-0.11	0.58
								(0.10,0.14)	(-0.14,-0.08)	(0.51,0.65)
Met need for family planning	2000	9306	24.41%	12.25%	36.64%	24.39%	2.99	0.12	0.09	0.64
								(0.18,0.24)	(0.04,0.14)	(0.52,0.76)
	2005	10164	40.42%	30.65%	54.75%	24.10%	1.79	0.11	-0.03	0.50
								(0.09,0.13)	(-0.06,0.003)	(0.39,0.61)
	2010	11439	50.96%	41.60%	55.40%	13.80%	1.33	0.06	-0.11	0.49
								(0.52,0.07)	(-0.14,-0.8)	(0.24,0.74)
Abortion with a skilled provider	2000	261	81.90%	58.60%	97.70%	39.10%	1.67	0.10	-0.03	0.49
								(0.06,0.14)	(-0.10,0.4)	(0.24,0.74)
	2005	617	78.40%	58.10%	89.50%	31.40%	1.54	0.07	-0.12	0.44
								(0.04,0.10)	(-0.22,-0.02)	(0.27,0.61)
	2010	2101	84.50%	78.92%	83.16%	4.24%	1.08	0.01	-0.23	0.46
								(-0.02,0.04)	(-0.29,-0.17)	(-0.01,0.93)

### Equity in service use

#### Equity gaps

**Figure 1:**
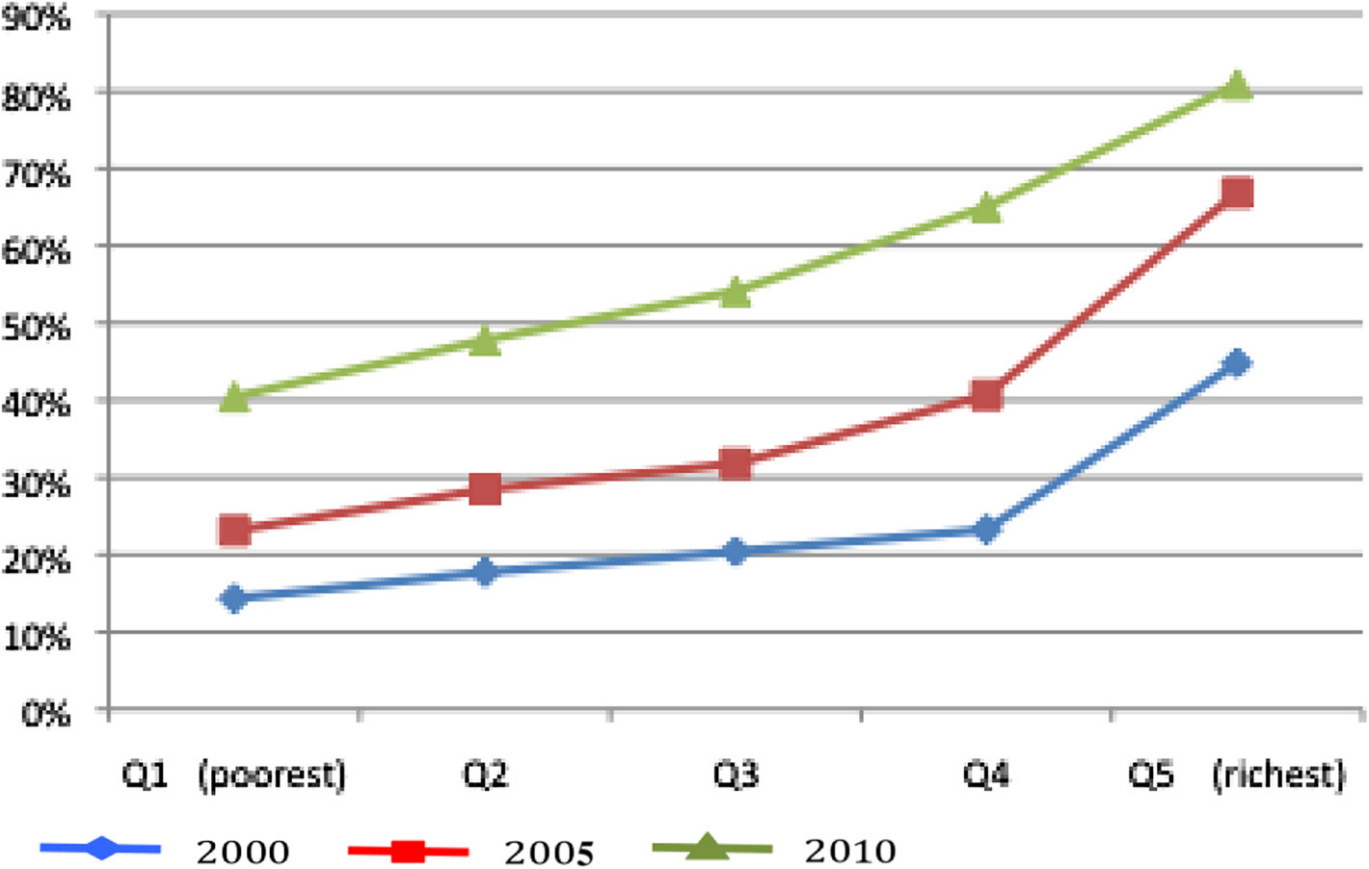
Composite coverage index of reproductive and maternal health service use by wealth quintile, Cambodia, 2000–2010.

Figure 1 graphs the composite coverage index by wealth quintile and year. This figure shows that utilisation across all services increased progressively each year. However, in absolute terms, utilisation increased the most for women in quintile 4 (second richest), closely followed by quintile 5 (richest). The percentage point increase in service use was smallest amongst women in quintiles 1 (poorest) and 2 (second poorest).

**Figure 2:**
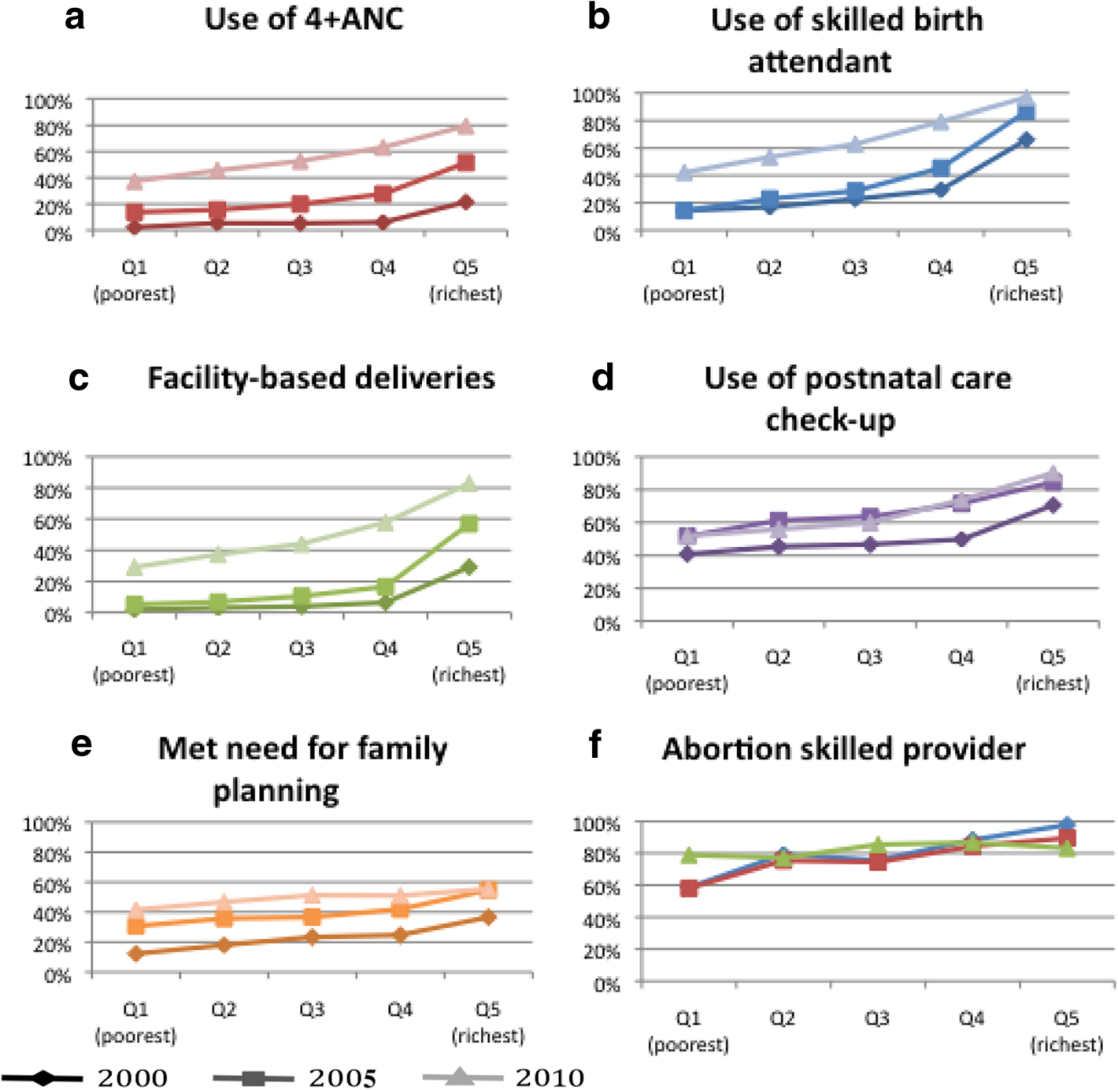
Reproductive and maternal health service use by wealth quintile, Cambodia, 2000–2010.

Similarly, the greatest absolute increases in maternal health service use (ANC, SBA, FBD, PNC) between 2000 and 2010 tended to be amongst the richer quintiles of the population, with the equity gap subsequently increasing over time for these services (Figure 2). Use of PNC services decreased for women in quintiles 2 and 3 between 2005 and 2010; increase in PNC use was almost double amongst the richest women compared to the poorest between 2000–2010. Met need for family planning and safe abortion were the only services where the equity gap in use between the richest and poorest quintiles decreased over time.

Figure 2a illustrates a positive wealth gradient in service use, whereby service use successively increases amongst richer quintiles compared to consecutively poorer quintiles. Use of at least four ANC visits, SBA, FBD and PNC in 2000 exhibited what some have coined ‘top inequality’, whereby the major difference in service use was between the top quintile and the rest of the population [12,26]. Over the last 10 years a shift is evident in the pattern of inequality in use of these four services, such that in 2010 wealth gradients now reflect a linear inequality, with service use progressively increasing through consecutive wealth quintiles from poorest to richest. Met need for family planning comprised a linear inequality pattern in 2000, with this virtually disappearing in 2010 (Figure 2e). Such changes in wealth gradient are less evident for use of a skilled provider for abortion (Figure 2f).

### Equity ratios

Equity ratios progressively declined between 2000–2010 for nearly all services, indicating that by this measure use has become more equitable over the last decade (Table 2). The greatest reduction in equity ratios was seen in FBD. Equity ratios remained approximately the same over the study period for use of PNC, suggesting that equity in service use has not improved for these services, however the overall level of inequity depicted by the ratio for PNC is relatively small.

### Concentration curves

**Figure 3:**
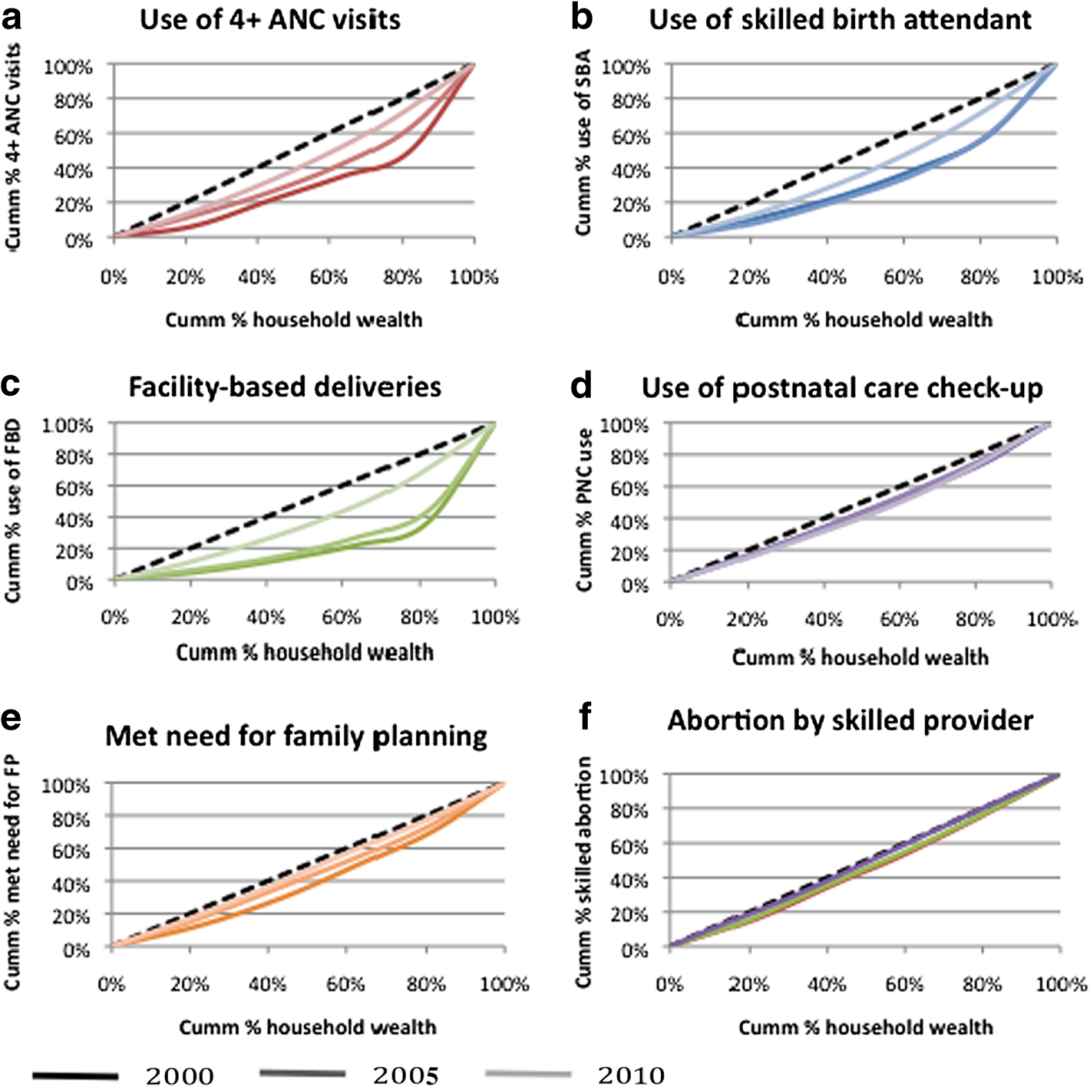
Concentration curves of reproductive and maternal health service use, Cambodia, 2000–2010.

Inspection of concentration curves shows that for all three years, and for all six services, there is inequity in use favouring the rich, i.e. services are used disproportionately more by wealthier women than by poorer women (Figure 3). However there is an impressive trend of clearly decreasing inequity over time, as the curves become shallower between 2000 and 2010, particularly for ANC and FBD. Within each year the ranking of services by level of inequity remains approximately the same, with FBD consistently the most inequitable service, depicted by the deepest curve, followed by SBA. Family planning, and abortion by skilled provider are consistently the shallowest curves in each year, illustrating that these services have the greatest equity in use. By 2010 the curves for both abortion by skilled provider and met need for family planning virtually follow the line of equality, reflecting almost perfect equity in service use across socio-economic groups.

### Concentration indices

Indirectly standardised concentration indices also clearly illustrate the striking improvements in equity in reproductive and maternal health service use over the decade (Table 2). The greatest decrease in indices was for FBD, which dropped from 0.58 to 0.22. Despite this improvement, inequality in FBD remained the highest of all six services in 2010, closely followed by SBA. The service with the least improvement in equity in use over the last decade was PNC, for which the index increased, indicating an increase in inequity. The service with the index closest to zero, indicating perfect equity in service use was skilled abortion provider, closely followed by met need for family planning.

Similar trends were found when estimates of the concentration index were disaggregated by urban and rural areas. Equity in service use has improved over the last decade in both types of residence (Table 2). However, inequity in service use was substantially higher within urban compared to rural populations, across all six services. Amongst rural populations in 2010 there was in fact increasing inequality favouring the *poor*, such that poorer groups are accessing services disproportionately more than wealthier groups, with concentration indices becoming increasingly negative.

### Robustness checks

Equity analysis using the household asset index was also conducted using a common set of assets in each year. Results of the comparative analysis of asset variables are presented in Additional file 2. Concentration indices produced using differing asset variables diverged from those calculated using common assets typically by only 0.01-0.02 of an index. This suggests that the results are robust to changes in asset variables used to compile the asset index. Similarly, equity estimates measured using either household asset indices or education as the social stratification variable produced qualitatively similar results (Additional file 1).

## Discussion

### Key findings

This study aimed to estimate equity in access to six reproductive and maternal health services in Cambodia over the last decade. The findings show that substantial progress has been made in Cambodia over the last 10 years, both in increasing population use of reproductive and maternal health services and in improving equity. Equity ratios, concentration curves and concentration indices indicate that inequity in service use has progressively decreased over time or remained stable at a low level for all services except PNC, which experienced a slight increase in inequity over the study period. Although FBD remained the service with the greatest inequity in 2010, it also saw the largest improvement in equity over time. Encouragingly, met need for family planning and use of abortion by skilled provider were found to be almost perfectly equitable.

Service coverage and equity in service use are inter-related concepts, whereby high levels of coverage reflect low inequity in service use, as services will be reached by most of the population, regardless of socio-economic group. However, generalised low coverage across all socio-economic groups *also* results in low inequity in service use, as most of the population have the same low access to care [53,54]. Therefore when interpreting equity analysis findings, it is important to be aware of overall coverage levels as well. Our results show both increases in overall coverage of service use and improvements in equity, suggesting that the former is not purely a consequence of increases in utilisation by the wealthiest.

The latest Cambodian DHS (2010) estimates a remarkable reduction in the maternal mortality ratio from 472 (CI 95%: 388, 605) deaths per 100,000 live births in 2005 to 206 (CI 95%: 124, 288) in 2010 [42]. Cambodia is reported to be on track to achieve its MDG5 target of 140 maternal deaths per 100,000 live births by 2015 [1]. This is an impressive achievement given Cambodia’s recent history of authoritarian rule and genocide experienced under the Khmer Rouge (1975–1979), followed by years of political unrest, which saw the MMR in 1990 at a staggering 830 per 100,000 live births [1]. The reduction in MMR is one of several key health indicators which the latest DHS indicates have improved in Cambodia including neonatal, infant and under five mortality, whilst overall increases in service coverage have occurred concomitantly with a drop in total fertility rate [42]. It is not possible to make any causal inferences about such improvements in health outcomes from the data available. However, it is noteworthy that these have occurred over the same period of time as the equity improvements in reproductive and maternal service utilisation reported in this paper.

### Study limitations

Before interpreting the findings, we note several limitations of the study. Firstly, in the absence of available data on household consumption, we used a household asset index as a proxy measure for household wealth. There remains debate regarding the reliability and validity of the asset index as a measure of household wealth [45,47,55,56]. Secondly, data on service use collected within the DHS can have a recall period of between 2–5 years. However data on household assets relate to the year of data collection. As the wealth-poverty spectrum is dynamic, changing easily overtime, there may be disparity between reported household assets at the time of the survey, and household assets at the time of the service use in question. Thirdly, analysis is limited to service utilisation, not health outcomes which are ultimately of interest, for example maternal mortality, total fertility rate, or abortion related complications. Fourthly, in this study it was not possible to look at changes in service use between the poor and non-poor over time, because the DHS does not contain data which can be used to construct a satisfactory measure of poverty. Finally, data on abortion reported in the DHS may be unreliable, both through under-reporting of abortions and also regarding where abortion services were sought. Collecting data on abortion is particularly challenging due to the sensitivity of the topic. Fetters et al. (2008) found in a nationally representative survey of public hospitals and health centres in Cambodia, reported abortions based on health facility records were 15 times greater than the DHS 2010 estimate [57]. Furthermore, research suggests Cambodian women often attempt multiple methods of abortion before going to a facility [58,59]. If more than one procedure and location has been used to abort a pregnancy, this may create errors and inconsistencies in the data.

### Study findings and the existing literature

These findings support those in the existing literature, which present evidence of substantial inequities in use of SBA and FBD by wealth [5-15] and education [6,7,11,16] favouring wealthier, more educated women. The general trend in the literature is also that urban women use reproductive and maternal health services more than rural women [10,11,14-18]. However using concentration indices with data disaggregated by urban/rural location, inequity in service use was actually dramatically higher within urban populations than amongst rural. Such estimates are not typically calculated in other studies. The findings support other studies which found evidence of inequity in ANC favouring wealthier, more educated women [5,6,8,9,14,21], and also the few studies to date that assess inequity in use of PNC, which find service use favouring wealthier, more educated women [6,11]. The findings concur with other studies in Asia which suggest that inequity in unmet need for family planning may be narrowing over time [9,25].

We are unaware of any other studies to date assessing trends in equity of reproductive and maternal health service use in Cambodia over time. The Countdown Equity Group (2008) included Cambodia in its assessment of equity in use of a group of health interventions in 54 countries, including family planning, ANC and SBA. However, this analysis produced a composite coverage index across all services, rather than considering specific trends for individual services. The Countdown study found that in Cambodia the coverage (equity) gap in the composite index decreased by 16.9 percentage points in Cambodia between 2000 and 2006. This supports the current finding of decreasing inequity in the six reproductive and maternal health services studied here [26]. Mohanty and Pathak (2009) assessed trends in at least three ANC visits during pregnancy, safe delivery and unmet need for family planning in India between 1992–2005 using three sets of survey data by estimating concentration indices. Inequity improved over time for ANC and safe delivery; however the extent of improvement in equity was substantially less than was found in Cambodia. Conversely, whilst in Cambodia equity in met need for family planning also improved over the decade, in India inequity in unmet need worsened over the time period studied.

### Explaining the findings

The improvements in coverage and equity of maternal health services observed in Cambodia may possibly be linked to a range of demand and supply side interventions implemented in the health sector over the study period, although given the lack of rigorous evidence, any discussion of these links should be considered tentative. Over the period of our analysis, the government and its partners made concerted efforts to address reproductive, maternal, newborn and child health. These include, on the demand-side, financing policies in the form of HEFs, vouchers and CBHI as well as, on the supply-side, midwifery training and incentive schemes [60]. At the same time, there have been general improvements in socioeconomic conditions that may have influenced health-related behaviours.

HEFs have expanded to 44 out of 77 operational districts since their introduction in 2000, and now cover approximately 2.5 million poor people [28,61]. They target the poor using the Ministry of Planning’s household identification system. Eligible households can then access free or subsidised tertiary healthcare, including reproductive and maternal care, for which providers are reimbursed by the Funds. A post-identification system also operates at the hospital level, whereby poor clients who seek care without a HEF card can be interviewed at the point of service use for eligibility to access free services [61-63].

HEFs have operated at sufficient scale to have a national impact. However, it is unclear to what extent the improvements described in this paper can be attributed to them. First, HEFs have largely focused on curative care, particularly hospitalisation, and not preventive services; and the package of services covered by HEFs was only recently expanded to include those provided by health centres, with only 28 percent of the latter covered at present. Yet it is this level of provider that mostly delivers ANC, PNC, and FP services. Second, evidence concerning the impact of HEFs is, at best, mixed. [64] Flores et al. (2011) find no significant impact on healthcare utilisation. Ir et al. (2010) show that a combination of vouchers, HEFs and performance-based incentives for providers was associated with increased FBD in one province in Cambodia, though their analysis does not enable them to attribute specific impact to individual interventions [65].

CBHI and vouchers targeting maternal health services have been implemented at insufficient scale to explain our findings, even in part. CBHI schemes started in Cambodia around the same time as HEFs, providing insurance for the non-poor informal sector. Enrolment is voluntary, with premiums typically less than $3 per family per month and benefits varying from scheme to scheme. Generally, they provide access to public health services and transport reimbursements for hospitals visits. However, CBHI has faced many challenges in Cambodia and currently they cover a mere 150,000 people (1% of the total population; 1.4% of the non-poor population) [61]. Vouchers targeting the poor for specific reproductive, maternal and child health services have been implemented in recent years only and on a pilot basis in three provinces [66].

Such demand-side interventions have been implemented against a backdrop of supply-side efforts to improve maternal, obstetric and newborn care in public facilities over the last five to 10 years. Training of midwives has been a government priority since 2000, and recent estimates suggest that all health centres now have at least one primary midwife and 51% have a secondary midwife [67]. The Royal Government of Cambodia’s (RCoG) midwifery incentive scheme was introduced in 2007, providing $15 to health centres and $10 to hospitals for every live birth, to encourage increased use of public services for deliveries and improved service quality [67]. Ir and Chheng (2012) provide suggestive evidence that the midwifery scheme may have contributed to increased SBA [68]. Concomitantly the government has banned deliveries by traditional midwives and strongly discourages home births attended by trained midwives [67]. While all these policies may plausibly have contributed to our findings to, the extent to which this is the case is unclear.

Developments outside the health sector such as improvements to infrastructure (e.g. roads, mobile phone coverage), education and the economy, ongoing in Cambodia for the last decade, are likely to also have a role in the trends observed. For example, the proportion of women aged 15–49 with no education has halved over the study period. Moreover, poverty has fallen while the economy has rapidly grown [35,42,44].

Although the overall picture in service coverage was clearly improving, there remains considerable room for improvement across all the services. Whereas met need for family planning stands out in the analysis as the most equitable service in 2010, *unmet* need for family planning remained at 17% of currently married women in Cambodia. Whilst modern contraceptive prevalence has increased from 18.5% of currently married women in 2000 to 34.9% in 2010 [42], this is still far from the MDG5 target of 60% by 2015 [28,69]. Furthermore, studies suggest there are persistent negative rumours and misinformation held amongst Cambodian women regarding the use of contraceptives, whilst reported experiences of negative side effects and improper use are common [58,59]. Unmarried sexually active women (excluded from the calculation of unmet need for family planning in the DHS) have been found in qualitative research to be particularly poorly catered for in accessing family planning services and information about contraception [58]. In addition, calculations of met need for family planning include women using both traditional and modern methods of contraception. More than one third of women currently reporting use of some form of contraception are using traditional methods in Cambodia [42]. It is likely that inequity in service use would be higher if met need for family planning was calculated for only those women using modern methods.

Abortion up to 12 weeks gestation was legalised in Cambodia in 1997. However there has since been slow progress in increasing use of safe abortion services [58,70]. Much work has been conducted under the National Reproductive Health Programme to improve the training and quality of service providers of abortion, and raise awareness of available services. However use of public abortion services remains low [58,70,71]. Since 2009 Mifepristone and Misoprosol have been more widely available in Cambodia, increasing access to medical abortion, which can be administered at home. Such policy improvements could have contributed to the decrease in inequity in use of skilled abortion providers found here, however as discussed above, substantial under-reporting and risk of errors in the data related to sources of abortion will also have influenced the estimates produced.

## Conclusion

Cambodia has made huge improvements in both coverage of reproductive and maternal health services and equity in use of these services over the last decade. Achieving improvements in maternal and reproductive health in practice requires attention to the distribution of service use and health outcomes within societies, as well as overall coverage rates. This study has highlighted the importance of equity analysis and the inadequacy of merely assessing aggregate coverage statistics. The growing literature on equity of access to health services in developing countries shows the persistence of inequities favouring wealthy, better-educated, urban populations. The findings in this study show that Cambodia has not escaped these trends, with disparities evident in 2010 in the use of four maternal health services between richer and poorer women; the greatest inequity found in use of FBD. However trends in equity of service use show that the direction of change is encouraging. Met need for family planning was found to be almost perfectly equitable in 2010. Such trends have been found in the context of supply side strengthening of the health system, large-scale implementation of a pro-poor health financing scheme, and general improvements in the socioeconomic conditions of the poor in Cambodia. The contribution of these factors to the trends observed in this paper should be the subject of further research. In particular, more rigorous evidence is needed on the impact of HEFs in relation to reproductive and maternal health services. This will be addressed in further quantitative and qualitative work to be conducted. It would also be interesting to explore whether similar equity trends are evident for other services such as those focusing on child health. As we strive for universal health coverage, future health policies and interventions must prioritise those services currently found to be most inequitable, specifically use of FBD and SBA, and also service use amongst poor urban populations.

## Abbreviations

ANC: Antenatal care; CBHI: Community-based health insurance; DHS: Demographic and health survey; FBD: Facility-based deliveries; HEF: Health equity fund; MDG: Millennium development goal; PNC: Postnatal care; RCoG: Royal Government of Cambodia; SBA: Skilled birth attendance.

## Competing interests

The authors declare they have no competing interests.

## Authors’ contributions

AD conducted the analysis, and wrote the first draft of the paper. TPJ and CG provided input into the design of the study as well as the analysis, and reviewed all drafts of the paper. All authors approved the final manuscript.

## Supplementary Material

Additional file 1 Description: Estimates of equity in reproductive and maternal health service use, Cambodia, 2000, 2005, and 2010 using household wealth and education social stratification variables. Click here for file

Additional file 2 Description: Estimates of equity in reproductive and maternal health service use, Cambodia, 2005 and 2010, using both common and maximum household assets to create the household wealth social stratification variable. Click here for file

Additional file 3 Description: Descriptive statistics for sample for each of six reproductive and maternal health service outcome variables, from Cambodia Demographic and Health Survey data 2000–2010. Click here for file
