# Supplementary material for: A decade of improvements in equity of access to reproductive and maternal health services in Cambodia, 2000–2010
Source: Int J Equity Health. 2013 Jul 9;12:51. doi: 10.1186/1475-9276-12-51 (PMC3723953; doi:10.1186/1475-9276-12-51)
Supplement: Additional file 1 — Description: Estimates of equity in reproductive and maternal health service use, Cambodia, 2000, 2005, and 2010 using household wealth and education social stratification variables. [file 1475-9276-12-51-S1.pdf]

Additional File 1

Table A1i: Equity in reproductive and maternal health services Cambodia, 2000, measured by multiple social stratification variables

Service	Overall % service use	Social stratification variable		% service use	Equity gap (Q5-Q1/most ed-least ed, % points)	Equity ratio§	Indirectly standardised concentration index (95% CI)
4+ Antenatal care	9.01%	Household assets	Q1 (poorest)	2.22%	19.25%	9.67	0.43 (0.16 , 0.70)
			Q2	5.40%			
			Q3	5.28%			
			Q4	6.05%			
			Q5 (richest)	21.47%			
		Education	0-3 years	4.10%	47.98%	12.70	0.42 (0.39 , 0.45)
			4-6 years	6.96%			
			7-9 years	19.20%			
			10-12 years	19.46%			
			13+ years	52.08%			
Skilled birth attendance	32.21%	Household assets	Q1 (poorest)	14.31%	51.84%	4.62	0.33 (0.29 , 0.37)
			Q2	16.88%			
			Q3	22.83%			
			Q4	29.48%			
			Q5 (richest)	66.15%			
		Education	0-3 years	18.25%	68.85%	4.77	0.26 (0.21 , 0.31)
			4-6 years	30.92%			
			7-9 years	53.74%			
			10-12 years	74.13%			
			13+ years	87.10%			
Facility-based delivery	10.04%	Household assets	Q1 (poorest)	1.82%	27.39%	16.05	0.58 (0.52 , 0.64)
			Q2	3.00%			
			Q3	3.88%			
			Q4	6.43%			
			Q5 (richest)	29.21%			
		Education	0-3 years	3.66%	54.40%	15.86	0.43 (0.36 , 0.50)
			4-6 years	7.97%			
			7-9 years	18.66%			
			10-12 years	41.15%			
			13+ years	58.06%			
Postnatal care	54.62%	Household assets	Q1 (poorest)	40.89%	29.64%	1.72	0.10 (0.08 , 0.12)
			Q2	45.31%			
			Q3	46.56%			
			Q4	49.62%			
			Q5 (richest)	70.53%			
		Education	0-3 years	45.42%	38.45%	1.85	0.09 (0.07 , 0.11)
			4-6 years	49.92%			
			7-9 years	60.68%			
			10-12 years	79.15%			
			13+ years	83.87%			
Met need for family planning	24.41%	Household assets	Q1 (poorest)	12.25%	24.39%	2.99	0.21 (0.18 , 0.24)
			Q2	17.97%			
			Q3	23.33%			
			Q4	24.70%			
			Q5 (richest)	36.64%			
		Education	0-3 years	18.08%	19.81%	2.10	0.12 (0.09 , 0.15)
			4-6 years	23.69%			
			7-9 years	28.99%			
			10-12 years	39.75%			
			13+ years	37.89%			
Abortion by skilled provider	81.88%	Household assets	Q1 (poorest)	58.62%	39.11%	1.67	0.10 (0.06 , 0.14)
			Q2	79.25%			
			Q3	75.56%			
			Q4	88.33%			
			Q5 (richest)	97.73%			
		Education	0-3 years	67.80%	32.20%	1.47	0.07 (0.03 , 0.11)
			4-6 years	86.81%			
			7-9 years	89.19%			
			10-12 years	100.00%			
			13+ years	100.00%			

§ Calculated as proportion of serivce use in richest wealth quintile/most educated group divided by proportion of service use in poorest quintile/least educated group

Table A1ii: Equity in reproductive and maternal health services Cambodia, 2005, measured by multiple social stratification variables

Service	Overall % service use	Social stratification variable		% service use	Equity gap (Q5-Q1/most ed-least ed, % points)	Equity ratio[§]	Indirectly standardised concentration index (95% CI)
**4+ Antenatal care**	26.98%	Household assets	Q1 (poorest)	13.78%	37.64%	3.73	0.28 (0.25 , 0.31)
			Q2	15.58%			
			Q3	19.85%			
			Q4	27.65%			
			Q5 (richest)	51.42%			
		Education	0-3 years	14.45%	64.96%	5.50	0.26 (0.23 , 0.29)
			4-6 years	25.88%			
			7-9 years	39.94%			
			10-12 years	55.17%			
			13+ years	79.41%			
**Skilled birth attendance**	43.76%	Household assets	Q1 (poorest)	14.36%	72.40%	6.04	0.35 (0.30 , 0.40)
			Q2	23.05%			
			Q3	28.68%			
			Q4	45.51%			
			Q5 (richest)	86.76%			
		Education	0-3 years	21.16%	72.44%	4.42	0.29 (0.26 , 0.32)
			4-6 years	42.12%			
			7-9 years	67.13%			
			10-12 years	81.69%			
			13+ years	93.60%			
**Facility-based delivery**	21.18%	Household assets	Q1 (poorest)	5.24%	51.55%	10.84	0.50 (0.43 , 0.57)
			Q2	6.78%			
			Q3	10.44%			
			Q4	16.62%			
			Q5 (richest)	56.79%			
		Education	0-3 years	8.45%	65.95%	8.80	0.38 (0.33 , 0.43)
			4-6 years	18.02%			
			7-9 years	35.09%			
			10-12 years	50.17%			
			13+ years	74.40%			
**Postnatal care**	69.87%	Household assets	Q1 (poorest)	53.98%	31.93%	1.59	0.09 (0.07 , 0.11)
			Q2	62.82%			
			Q3	66.31%			
			Q4	73.61%			
			Q5 (richest)	85.91%			
		Education	0-3 years	61.24%	34.84%	1.57	0.06 (0.04 , 0.08)
			4-6 years	68.41%			
			7-9 years	79.21%			
			10-12 years	85.34%			
			13+ years	96.08%			
**Met need for family planning**	40.42%	Household assets	Q1 (poorest)	30.65%	24.10%	1.79	0.11 (0.09 , 0.13)
			Q2	35.77%			
			Q3	36.64%			
			Q4	41.93%			
			Q5 (richest)	54.75%			
		Education	0-3 years	32.34%	22.00%	1.68	0.11 (0.09 , 0.13)
			4-6 years	42.53%			
			7-9 years	48.41%			
			10-12 years	54.18%			
			13+ years	54.34%			
**Abortion by skilled provider**	78.36%	Household assets	Q1 (poorest)	58.12%	31.35%	1.54	0.07 (0.04 , 0.10)
			Q2	75.38%			
			Q3	74.44%			
			Q4	84.33%			
			Q5 (richest)	89.47%			
		Education	0-3 years	69.36%	16.35%	1.24	0.06 (0.03 , 0.09)
			4-6 years	77.00%			
			7-9 years	79.31%			
			10-12 years	97.22%			
			13+ years	85.71%			

[§] Calculated as proportion of serivce use in richest wealth quintile/most educated group divided by proportion of service use in poorest quintile/least educated group

Table A1iii: Equity in reproductive and maternal health services Cambodia, 2010, measured by multiple socio-economic status variables

Service	Overall % service use	Social stratification variable		% service use	Equity gap (Q5-Q1/most ed-least ed, % points)	Equity ratio§	Indirectly standardised concentration index (95% CI)
4+ Antenatal care	57.27%	Household assets	Q1 (poorest)	37.37%	42.10%	2.13	0.15 (0.13 , 0.17)
			Q2	45.62%			
			Q3	52.73%			
			Q4	63.25%			
			Q5 (richest)	79.47%			
		Education	0-3 years	38.28%	51.76%	2.35	0.15 (0.13 , 0.17)
			4-6 years	57.26%			
			7-9 years	70.48%			
			10-12 years	79.03%			
			13+ years	90.04%			
Skilled birth attendance	68.81%	Household assets	Q1 (poorest)	42.23%	54.57%	2.29	0.17 (0.15 , 0.19)
			Q2	53.42%			
			Q3	62.95%			
			Q4	79.32%			
			Q5 (richest)	96.80%			
		Education	0-3 years	47.75%	51.05%	2.07	0.11 (0.09 , 0.11)
			4-6 years	70.61%			
			7-9 years	85.10%			
			10-12 years	95.43%			
			13+ years	98.80%			
Facility-based delivery	53.07%	Household assets	Q1 (poorest)	29.16%	53.69%	2.84	0.22 (0.20 , 0.24)
			Q2	37.34%			
			Q3	43.98%			
			Q4	57.73%			
			Q5 (richest)	82.85%			
		Education	0-3 years	33.94%	56.15%	2.65	0.19 (0.16 , 0.21)
			4-6 years	51.69%			
			7-9 years	65.74%			
			10-12 years	78.50%			
			13+ years	90.09%			
Postnatal care	73.84%	Household assets	Q1 (poorest)	51.81%	38.36%	1.74	0.12 (0.10 , 0.14)
			Q2	55.83%			
			Q3	59.78%			
			Q4	73.83%			
			Q5 (richest)	90.17%			
		Education	0-3 years	50.40%	44.65%	1.89	0.12 (0.10 , 0.14)
			4-6 years	69.60%			
			7-9 years	79.06%			
			10-12 years	87.14%			
			13+ years	95.05%			
Met need for family planning	50.96%	Household assets	Q1 (poorest)	41.60%	13.80%	1.33	0.06 (0.05 , 0.07)
			Q2	46.75%			
			Q3	51.29%			
			Q4	51.03%			
			Q5 (richest)	55.40%			
		Education	0-3 years	42.12%	13.93%	1.33	0.06 (0.05 , 0.07)
			4-6 years	49.94%			
			7-9 years	54.68%			
			10-12 years	57.57%			
			13+ years	56.05%			
Abortion by skilled provider	84.48%*	Household assets	Q1 (poorest)	77.26%	6.43%	1.08	0.01 (-0.02 ,0.04)
			Q2	79.64%			
			Q3	84.84%			
			Q4	85.65%			
			Q5 (richest)	83.69%			
		Education	0-3 years	78.55%	6.64%	1.08	0.03 (0.00 , 0.06)
			4-6 years	83.64%			
			7-9 years	88.58%			
			10-12 years	84.72%			
			13+ years	85.19%			

§ Calculated as proportion of serivce use in richest wealth quintile/most educated group divided by proportion of service use in poorest quintile/least educated group

* Excludes all women who report having a medical abortion, which can be administered at home
