# Supplementary material for: A decade of improvements in equity of access to reproductive and maternal health services in Cambodia, 2000–2010
Source: Int J Equity Health. 2013 Jul 9;12:51. doi: 10.1186/1475-9276-12-51 (PMC3723953; doi:10.1186/1475-9276-12-51)
Supplement: Additional file 2 — Description: Estimates of equity in reproductive and maternal health service use, Cambodia, 2005 and 2010, using both common and maximum household assets to create the household wealth social stratification variable. [file 1475-9276-12-51-S2.pdf]

Additional File 2

Table A2i: Equity in use of reproductive and maternal health services in Cambodia by common and maximum household assets, 2005

Service	Overall % service use		% service use by wealth quintile (max assets)	% service use by wealth quintile (common assets)	Equity gap (Q5-Q1, % points) (max assets)	Equity gap (Q5-Q1, % points) (common assets)	Equity ratio (max assets) §	Equity ratio (common assets) §	Indirectly standardised concentration index (max assets)	Indirectly standardised concentration index (common assets)
4+ Antenatal care	26.98%	Q1 (poorest)	13.78%	12.57%	37.64%	38.96%	3.73	4.10	0.28 (0.25 , 0.31)	0.29 (0.26 , 0.32)
		Q2	15.58%	16.11%						
		Q3	19.85%	20.99%						
		Q4	27.65%	27.81%						
		Q5 (richest)	51.42%	51.53%						
Skilled birth attendance	43.76%	Q1 (poorest)	14.36%	14.64%	72.40%	72.41%	6.04	5.95	0.35 (0.30 , 0.40)	0.35 (0.30 , 0.40)
		Q2	23.05%	22.67%						
		Q3	28.68%	30.62%						
		Q4	45.51%	45.05%						
		Q5 (richest)	86.76%	87.05%						
Facility based delivery	21.18%	Q1 (poorest)	5.24%	5.47%	51.55%	51.42%	10.84	10.40	0.5 (0.43 , 0.57)	0.46 (0.40 , 0.52)
		Q2	6.78%	7.23%						
		Q3	10.44%	10.83%						
		Q4	16.62%	16.34%						
		Q5 (richest)	56.79%	56.89%						
Postnatal care	67.57%	Q1 (poorest)	53.98%	55.64%	31.93%	30.52%	1.59	1.55	0.09 (0.07 , 0.11)	0.09 (0.07 , 0.11)
		Q2	62.82%	63.54%						
		Q3	66.31%	65.55%						
		Q4	73.61%	72.09%						
		Q5 (richest)	85.91%	86.16%						
Met need for family planning	40.42%	Q1 (poorest)	30.65%	29.32%	24.10%	25.18%	1.79	1.86	0.11 (0.09 , 0.13)	0.12 (0.10 , 0.14)
		Q2	35.77%	35.20%						
		Q3	36.64%	38.77%						
		Q4	41.93%	42.41%						
		Q5 (richest)	54.75%	54.50%						
Abortion with a skilled provider	78.36%	Q1 (poorest)	58.12%	62.30%	31.35%	27.70%	1.54	1.44	0.07 (0.04 , 0.10)	0.07 (0.04 , 0.10)
		Q2	75.38%	73.17%						
		Q3	74.44%	71.74%						
		Q4	84.33%	85.38%						
		Q5 (richest)	89.47%	90.00%						

§ Calculated as proportion of service use in richest wealth quintile divided by proportion of service use in poorest quintile

Table A2ii: Equity in use of reproductive and maternal health services in Cambodia by common and maximum household assets, 2010

Service	Overall % service use		% service use stratified by SES (max assets)	% service use stratified by SES (common assets)	Equity gap (Q5-Q1, % points) (max assets)	Equity gap (Q5-Q1, % points) (common assets)	Equity ratio (max assets) §	Equity ratio (common assets) §	Indirectly standardised concentration index (max assets)	Indirectly standardised concentration index (common assets)
**4+ Antenatal care**	57.27%	Q1 (poorest)	37.37%	35.58%	42.10%	44.05%	2.13	2.24	0.15 (0.13 , 0.17)	0.16 (0.14 , 0.18)
		Q2	45.62%	46.01%						
		Q3	52.73%	51.60%						
		Q4	63.25%	66.91%						
		Q5 (richest)	79.47%	79.63%						
**Skilled birth attendance**	68.81%	Q1 (poorest)	42.23%	42.16%	54.57%	54.72%	2.29	2.30	0.17 (0.15 , 0.19)	0.17 (0.15 , 0.19)
		Q2	53.42%	51.92%						
		Q3	62.95%	64.29%						
		Q4	79.32%	81.20%						
		Q5 (richest)	96.80%	96.88%						
**Facility based delivery**	53.07%	Q1 (poorest)	29.16%	30.21%	53.69%	52.36%	2.84	2.73	0.22 (0.20 , 0.24)	0.22 (0.20 , 0.24)
		Q2	37.34%	34.48%						
		Q3	43.98%	44.94%						
		Q4	57.73%	60.08%						
		Q5 (richest)	82.85%	82.57%						
**Postnatal care**	73.84%	Q1 (poorest)	51.81%	49.45%	38.36%	40.88%	1.74	1.83	0.12 (0.10 , 0.14)	0.13 (0.11 , 0.15)
		Q2	55.83%	54.25%						
		Q3	59.78%	63.23%						
		Q4	73.83%	75.64%						
		Q5 (richest)	90.17%	90.33%						
**Met need for family planning**	50.96%	Q1 (poorest)	41.60%	39.95%	13.80%	15.34%	1.33	1.38	0.06 (0.05 , 0.07)	0.06 (0.05 , 0.07)
		Q2	46.75%	49.02%						
		Q3	51.29%	48.94%						
		Q4	51.03%	52.04%						
		Q5 (richest)	55.40%	55.29%						
**Abortion with a skilled provider**	84.48%*	Q1 (poorest)	77.26%	76.90%	6.43%	6.09%	1.08	1.08	0.01 (-0.02 ,0.04)	0.01 (-0.02 , 0.04)
		Q2	79.64%	80.06%						
		Q3	84.84%	85.95%						
		Q4	85.65%	86.56%						
		Q5 (richest)	83.69%	82.99%						

§ Calculated as proportion of service use in richest wealth quintile divided by proportion of service use in poorest quintile

* Excludes all women who report having a medical abortion, which can be administered at home
