# Supplementary material for: A decade of improvements in equity of access to reproductive and maternal health services in Cambodia, 2000–2010
Source: Int J Equity Health. 2013 Jul 9;12:51. doi: 10.1186/1475-9276-12-51 (PMC3723953; doi:10.1186/1475-9276-12-51)
Supplement: Additional file 3 — Description: Descriptive statistics for sample for each of six reproductive and maternal health service outcome variables, from Cambodia Demographic and Health Survey data 2000–2010. [file 1475-9276-12-51-S3.pdf]

Table A3

Table A3i: Descriptive statistics, women using antenatal care, Cambodia, DHS 2000-2010

Year	2000		2005		2010	
Variable	Mean	Standard deviation	Mean	Standard deviation	Mean	Standard deviation
Age (years)	31.15	6.98	30.13	7.18	29.32	6.60
Age at delivery (years)	29.57	6.75	28.52	6.9	27.63	6.31
Age at first marriage (years)	19.31	3.76	19.46	3.78	19.74	3.66
Parity (births)	3.89	2.49	3.23	2.21	2.68	1.84
Household size (people)	6.11	2.20	5.70	2.22	5.60	2.15
Urban residence	0.14	0.34	0.14	0.35	0.16	0.37
**Highest level of education**						
No education	0.32	0.47	0.23	0.42	0.17	0.38
Primary	0.54	0.50	0.59	0.49	0.56	0.50
Secondary	0.14	0.35	0.17	0.38	0.25	0.43
Higher	0.002	0.04	0.01	0.07	0.02	0.13
**Religion**						
Buddhist	0.96	0.19	0.97	0.18	0.97	0.18
Muslim	0.02	0.15	0.02	0.13	0.02	0.13
Christian	0.002	0.05	0.004	0.07	0.00	0.06
Other	0.01	0.12	0.01	0.11	0.01	0.11
**Marital status**						
Never married	0	0	0.00	0.02	0.00	0.02
Living together	-	-	0.003	0.06	0.01	0.08
Married	0.94	0.24	0.94	0.23	0.95	0.22
Widowed	0.04	0.19	0.03	0.18	0.02	0.19
Divorced	0.02	0.14	0.03	0.18	0.04	0.23
Not living together	0.01	0.08	0.02	0.16	0.02	0.17
**Husband's occupation**						
Did not work	0.02	0.16	-	-	0.00	0.06
Professional/technician/manager	0.07	0.26	0.04	0.21	0.08	0.26
Clerical	0.01	0.09	0.02	0.14	0.01	0.12
Sales	0.04	0.19	0.06	0.24	0.06	0.23
Agricultural self-employed	-	-	0.50	0.50	0.54	0.50
Agricultural employee	0.71	0.45	0.12	0.32	-	-
Services	0.01	0.11	0.05	0.21	0.05	0.22
Skilled manual	0.09	0.29	0.12	0.32	0.25	0.43
Unskilled manual	0.04	0.20	0.09	0.29	0.01	0.09
At least 4 ANC visits during pregnancy	0.09	0.29	0.27	0.44	0.60	0.49

N (2000) = 6049; N (2005) = 6075; N (2010) = 6371

Table A3ii: Descriptive statistics, women with skilled birth attendance at delivery, Cambodia, DHS 2000-2010

Year	2000		2005		2010	
Variable	Mean	Standard deviation	Mean	Standard deviation	Mean	Standard deviation
Age (years)	31.03	6.74	29.92	6.96	29.22	6.41
Age at delivery (years)	28.98	6.64	27.94	6.81	27.22	6.22
Age at first marriage (years)	19.44	3.74	19.42	3.74	19.71	3.67
Parity (births)	4.1	2.49	3.38	2.20	2.84	1.88
Household size (people)	6.17	2.18	5.80	2.23	5.67	2.18
Urban residence	0.13	0.34	0.14	0.35	0.16	0.36
**Highest level of education**						
No education	0.33	0.47	0.24	0.43	0.18	0.39
Primary	0.54	0.5	0.59	0.49	0.57	0.50
Secondary	0.14	0.34	0.16	0.37	0.23	0.42
Higher	0.002	0.04	0.01	0.07	0.02	0.12
**Religion**						
Buddhist	0.96	0.19	0.96	0.19	0.97	0.18
Muslim	0.02	0.14	0.02	0.13	0.05	0.12
Christian	0.002	0.05	0.004	0.07	0.00	0.06
Other	0.02	0.12	0.01	0.12	0.01	0.12
**Marital status**						
Never married	0	0	0.001	0.02	0.00	0.02
Living together	-	-	0.004	0.06	0.01	0.08
Married	0.95	0.22	0.95	0.22	0.95	0.21
Widowed	0.03	0.17	0.02	0.18	0.02	0.19
Divorced	0.02	0.13	0.03	0.18	0.04	0.23
Not living together	0.01	0.08	0.02	0.16	0.02	0.17
**Husband's occupation**						
Did not work	0.02	0.16	-	-	0.00	0.06
Professional/technician/manager	0.07	0.25	0.04	0.20	0.07	0.26
Clerical	0.01	0.09	0.02	0.14	0.01	0.11
Sales	0.04	0.19	0.06	0.24	0.06	0.23
Agricultural self-employed	-	-	0.50	0.50	0.55	0.50
Agricultural employee	0.72	0.45	0.12	0.33	-	-
Services	0.01	0.11	0.04	0.21	0.05	0.22
Skilled manual	0.09	0.28	0.12	0.32	0.25	0.43
Unskilled manual	0.04	0.19	0.09	0.29	0.01	0.09
Skilled birth attendance during delivery	0.32	0.47	0.44	0.50	0.71	0.45

N (2000) = 8729; N (2005) = 8201; N (2010) = 8115

Table A3iii: Descriptive statistics, women with facility based delivery, Cambodia, DHS 2000-2010

Year	2000		2005		2010	
**Variable**	**Mean**	**Standard deviation**	**Mean**	**Standard deviation**	**Mean**	**Standard deviation**
Age (years)	31.03	6.74	29.92	6.96	29.22	6.41
Age at delivery (years)	28.98	6.64	29.88	6.94	27.22	6.22
Age at first marriage (years)	19.43	3.74	19.42	3.74	19.65	3.72
Parity (births)	4.11	2.49	3.38	2.20	2.84	1.88
Household size (people)	6.17	2.18	5.80	2.23	5.68	2.18
Urban residence	0.13	0.34	0.14	0.35	0.16	0.36
**Highest level of education**						
No education	0.33	0.47	0.24	0.43	0.18	0.39
Primary	0.53	0.50	0.59	0.49	0.56	0.50
Secondary	0.13	0.34	0.16	0.37	0.23	0.42
Higher	0.00	0.04	0.01	0.07	0.01	0.12
**Religion**						
Buddhist	0.96	0.19	0.96	0.19	0.97	0.18
Muslim	0.02	0.14	0.02	0.13	0.01	0.12
Christian	0.00	0.05	0.00	0.07	0.00	0.06
Other	0.02	0.12	0.14	0.12	0.01	0.12
**Marital status**						
Never married	0.00	0.00	0.00	0.02	0.00	0.02
Living together	-	-	0.00	0.06	0.01	0.08
Married	0.95	0.22	0.95	0.22	0.95	0.21
Widowed	0.03	0.17	0.02	0.18	0.02	0.19
Divorced	0.02	0.13	0.03	0.18	0.04	0.23
Not living together	0.01	0.08	0.02	0.16	0.02	0.17
**Husband's occupation**						
Did not work	0.02	0.16	-	-	0.00	0.06
Professional/technician/manager	0.07	0.25	0.04	0.20	0.07	0.26
Clerical	0.01	0.09	0.02	0.14	0.01	0.11
Sales	0.04	0.19	0.06	0.24	0.05	0.23
Agricultural self-employed	-	-	0.50	0.50	0.55	0.50
Agricultural employee	0.72	0.45	0.12	0.33	-	-
Services	0.01	0.11	0.04	0.21	0.05	0.22
Skilled manual	0.09	0.28	0.12	0.32	0.25	0.43
Unskilled manual	0.04	0.19	0.09	0.29	0.01	0.09
Facility based delivery	0.10	0.30	0.21	0.41	0.53	0.50

N (2000) = 8746; N (2005) = 8201; N (2010) = 8138

Table A3iv: Descriptive statistics, women receiving postnatal care, Cambodia, DHS 2000-2010

Year	2000		2005		2010	
**Variable**	**Mean**	**Standard deviation**	**Mean**	**Standard deviation**	**Mean**	**Standard deviation**
Age (years)	31.03	6.75	30.13	7.18	29.34	6.61
Age at delivery (years)	28.98	6.64	28.52	6.90	27.64	6.32
Age at first marriage (years)	19.43	3.74	19.46	3.78	19.74	3.66
Parity (births)	4.11	2.49	3.23	2.21	12.69	1.84
Household size (people)	6.17	2.18	5.70	2.22	5.60	2.15
Urban residence	0.13	0.34	0.14	0.35	0.16	0.37
**Highest level of education**						
No education	0.33	0.47	0.23	0.42	0.17	0.38
Primary	0.53	0.50	0.59	0.49	0.56	0.50
Secondary	0.13	0.34	0.17	0.38	0.25	0.43
Higher	0.00	0.04	0.01	0.07	0.02	0.13
**Religion**						
Buddhist	0.96	0.19	0.97	0.18	0.97	0.18
Muslim	0.02	0.14	0.02	0.13	0.02	0.13
Christian	0.00	0.05	0.0	0.07	0.00	0.06
Other	0.02	0.12	0.01	0.11	0.01	0.11
**Marital status**						
Never married	0.00	0.00	0.00	0.02	0.00	0.02
Living together	-	-	0.00	0.06	0.01	0.08
Married	0.95	0.22	0.94	0.23	0.95	0.22
Widowed	0.03	0.17	0.03	0.18	0.02	0.19
Divorced	0.02	0.13	0.03	0.18	0.04	0.23
Not living together	0.01	0.08	0.02	0.16	0.02	0.17
**Husband's occupation**						
Did not work	0.02	0.16	-	-	0.00	0.06
Professional/technician/manager	0.07	0.25	0.04	0.21	0.08	0.26
Clerical	0.01	0.09	0.02	0.14	0.01	0.12
Sales	0.04	0.19	0.06	0.24	0.06	0.23
Agricultural self-employed	-	-	0.5	0.5	0.54	0.50
Agricultural employee	0.72	0.45	0.12	0.32	-	-
Services	0.01	0.11	0.05	0.21	0.05	0.22
Skilled manual	0.09	0.28	0.12	0.32	0.25	0.43
Unskilled manual	0.04	0.19	0.09	0.29	0.01	0.09
Postnatal care visit after delivery	0.55	0.50	0.70	0.46	0.74	0.44

N (2000) = 8737; N (2005) = 6076; N (2010) = 6374

Table A3v: Descriptive statistics, currently married women, Cambodia, DHS 2000-2010

Year	2000		2005		2010	
**Variable**	**Mean**	**Standard deviation**	**Mean**	**Standard deviation**	**Mean**	**Standard deviation**
Age (years)	33.5	8.32	33.6	8.69	33.34	8.66
Age at first marriage (years)	19.27	3.8	19.47	3.75	19.67	3.79
Household size (people)	6.05	2.16	5.67	2.19	5.46	2.08
Urban residence	0.16	0.36	0.15	0.36	1.82	0.38
**Highest level of education**						
No education	0.35	0.48	0.23	0.42	0.19	0.39
Primary	0.53	0.50	0.59	0.49	0.56	0.50
Secondary	0.12	0.33	0.17	0.38	0.24	0.43
Higher	0.002	0.04	0.01	0.08	0.01	0.12
**Religion**						
Buddhist	0.92	0.26	0.97	0.17	0.97	0.16
Muslim	0.02	0.15	0.02	0.13	0.01	0.12
Christian	0.002	0.05	0.00	0.06	0.00	0.06
Other	0.05	0.22	0.01	0.10	0.01	0.10
**Husband's occupation**						
Did not work	0.02	0.15	-	-	0.01	0.09
Professional/technician/manager	0.10	0.30	0.06	0.24	0.08	0.28
Clerical	0.01	0.10	0.02	0.15	0.02	0.12
Sales	0.04	0.20	0.07	0.25	0.06	0.24
Agricultural self-employed	-	-	0.49	0.50	0.55	0.50
Agricultural employee	0.70	0.46	0.11	0.32	-	-
Services	0.01	0.1	0.05	0.22	0.06	0.24
Skilled manual	0.09	0.28	0.11	0.31	0.22	0.41
Unskilled manual	0.03	0.18	0.08	0.27	0.01	0.09
Met neet for family planning	0.24	0.43	0.40	0.49	0.51	0.50

N (2000) = 9306; N (2005) = 10164; N (2010) = 11439

Table A3vi: Descriptive statistics, women reporting an abortion, Cambodia, DHS 2000-2010

Year	2000		2005		2010	
**Variable**	**Mean**	**Standard deviation**	**Mean**	**Standard deviation**	**Mean**	**Standard deviation**
Age (years)	34.84	6.83	32.62	7.16	36.25	7.07
Age at first marriage (years)	19.56	3.97	19.07	3.8	18.94	3.54
Household size (people)	6.08	2.01	5.62	2.46	5.60	2.01
Urban residence	0.20	0.40	0.25	0.44	0.2	0.4
**Highest level of education**						
No education	0.27	0.45	0.21	0.41	0.18	0.39
Primary	0.60	0.49	0.58	0.49	0.67	0.47
Secondary	0.13	0.33	0.21	0.41	0.15	0.35
Higher	0.00	0.00	0.00	0.06	0.00	0.06
**Religion**						
Buddhist	0.97	0.15	0.99	0.12	0.99	0.10
Muslim	0.02	0.13	0.01	0.08	0.01	0.09
Christian	0.01	0.08	0.01	0.09	0.00	0.02
Other	0.00	0.04	0.00	0.00	0.00	0.02
**Marital status**						
Never married	0.00	0.00	0.00	0.00	0.00	0.00
Living together	-	-	0.01	0.12	0.97	0.18
Married	0.95	0.21	0.94	0.25	0.01	0.08
Widowed	0.02	0.15	0.04	0.26	0.02	0.18
Divorced	0.02	0.15	0.04	0.26	0.03	0.20
Not living together	0.00	0.00	0.06	0.29	0.01	0.15
**Husband's occupation**						
Did not work	0.03	0.18	-	-	0.01	0.08
Professional/technician/manager	0.15	0.36	0.07	0.25	0.05	0.21
Clerical	0.00	0.07	0.05	0.21	0.01	0.12
Sales	0.08	0.27	0.06	0.23	0.05	0.21
Agricultural self-employed	-	-	0.34	0.47	0.51	0.50
Agricultural employee	0.47	0.50	0.11	0.31	-	-
Services	0.02	0.15	0.12	0.33	0.08	0.26
Skilled manual	0.16	0.37	0.17	0.37	0.29	0.45
Unskilled manual	0.09	0.28	0.09	0.28	0.01	0.11
Abortion with skilled provider	0.82	0.39	0.78	0.41	0.84	0.36

N (2000) = 261; N (2005) = 617; N (2010) = 2101
